# Cardiac Tamponade as a Rare Presentation of Adult-Onset Still's Disease

**DOI:** 10.7759/cureus.20147

**Published:** 2021-12-03

**Authors:** Shadi Daoud, Lean Alkhatib, Aseel Nimri, Ahmad S Matarneh

**Affiliations:** 1 Rheumatology, Royal Medical Services, Amman, JOR; 2 Internal Medicine, Royal Medical Services, Amman, JOR; 3 Pediatrics, Royal Medical Services, Amman, JOR; 4 Internal Medicine, Hamad Medical Corporation, Doha, QAT

**Keywords:** systemic disease, biologic treatment, internal medicine and rheumatology, pericardial tamponde, adult onset still's disease (aosd)

## Abstract

Adult-onset Still's disease (AOSD) is a rare inflammatory disease that affects multiple organ systems. Efforts have been made to study the course of the illness and possible treatment options. Cardiac tamponade is a rare and life-threatening complication of AOSD that can be the initial presentation of the disease.

We report a 34-year-old patient who presented with a picture of cardiac tamponade and underwent emergency pericardiocentesis. Upon further investigations, the diagnosis of AOSD was made based on Yamaguchi criteria. Furthermore, he showed significant improvement following treatment with prednisolone, methotrexate, and tocilizumab.

Our case provides evidence that AOSD should be considered in the differential diagnosis of cardiac tamponade and how prompt treatment of AOSD can effectively prevent potentially fatal complications.

## Introduction

Adult-onset Still's disease (AOSD) is an uncommon inflammatory illness that affects multiple organ systems [1]. It is a disease of unknown origin that mainly affects patients between the ages of 16 and 35. AOSD is characterized by prolonged fever, salmon-colored pink rash, arthritis, and other systemic manifestations [1,2]. One of the extra-articular presentations is pericarditis which can be rarely complicated with cardiac tamponade [3]. AOSD is diagnosed by applying the Yamaguchi criteria and ruling out other infectious, neoplastic, and autoimmune causes [4]. Steroids, disease-modifying anti-rheumatic drugs (DMARDs), and cyclosporine are the mainstay treatment options. Persistent cases can be managed by biologic DMARDs such as interleukin (IL)-6 antagonist tocilizumab [4,5]. Here, we present a rare case of cardiac tamponade as a presentation of AOSD that was efficiently managed by prednisolone, methotrexate, and tocilizumab.

## Case presentation

A thirty-four-year-old male patient with no previously known medical illnesses presented to our hospital with a one-day history of pleuretic chest pain and shortness of breath. He was also complaining of intermittent spikes of fever, bilateral joint pain in his hands, knees, and ankles, and a pink rash on his arms for about three weeks before his presentation. He had no previous similar symptoms and no history of upper respiratory tract symptoms.

On physical examination, vital signs showed hypotension (blood pressure of 80/50), tachycardia (heart rate of 120), tachypnea (respiratory rate of 20/minute), a body temperature of 37 Celsius, and oxygen saturation of 97% at room air. In addition, he was found to have distant heart sounds, jugular venous distention, splenomegaly, and a salmon-colored rash was noted on his upper limbs; his respiratory examination was normal.

Bedside transthoracic echocardiogram (TTE) showed circumferential pericardial effusion (maximal diameter, 22 mm) and collapse of the right atrium. Immediate pericardiocentesis was performed, draining a large amount of serosanguinous fluid.

During hospitalization, his complete blood count showed leukocytosis (WBC 16,000/uL with 88% neutrophils), hemoglobin of 11 gm/dl, and platelet count of 432,000/uL. His inflammatory markers were high (C-reactive protein (CRP) 48 mg/L, erythrocyte sedimentation rate (ESR) 90 mm/hr). He had hyperferritinemia 1539 ng/ml (28-356ng/ml) and a positive D-dimer. His anti-nuclear antibody (ANA) and rheumatic fever (RF) were negatives (Table 1).

**Table 1:** Basic laboratory investigations WBC - white blood cell, Hgb - hemoglobin, PLT - platelet, CRP - C-reactive protein, ESR - erythrocyte sedimentation rate, ALT - alanine transaminase, AST - aspartate aminotransferase, ALP - alkaline phosphatase

Laboratory	Result	Reference range
WBC	16x10^3/ul	4.5 – 5.5 x10^3/ul
Hgb	12 gm/dl	13-17 gm/dl
PLT	432x10^3/ul	165-415x10^3/ul
CRP	48 mg/L	0.3-10 mg/L
ESR	90 mm/hr	0-22 mm/hr
Ferritin	1539 ng/ml	28-356 ng/ml
ALT	43 U/L	0-41 U/L
AST	26 U/L	0-37 U/L
ALP	101 U/L	40-129 U/L
Triglycerides	80 mg/dl	50-200 mg/dl
Cholesterol	164 mg/dl	120-200 mg/dl

Blood, urine, and pericardial fluid cultures were negative. In addition, his purified protein derivative skin test (PPD) was negative.

**Figure 1:**
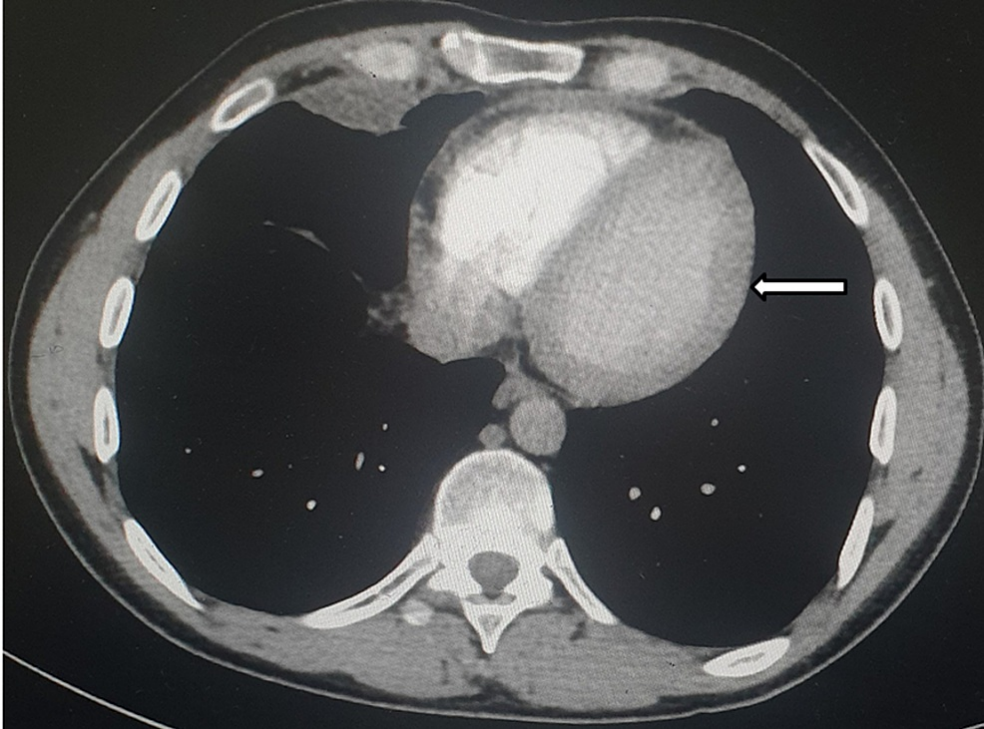
CT scan demonstrating mild pericardial effusion (white arrow)

**Figure 2:**
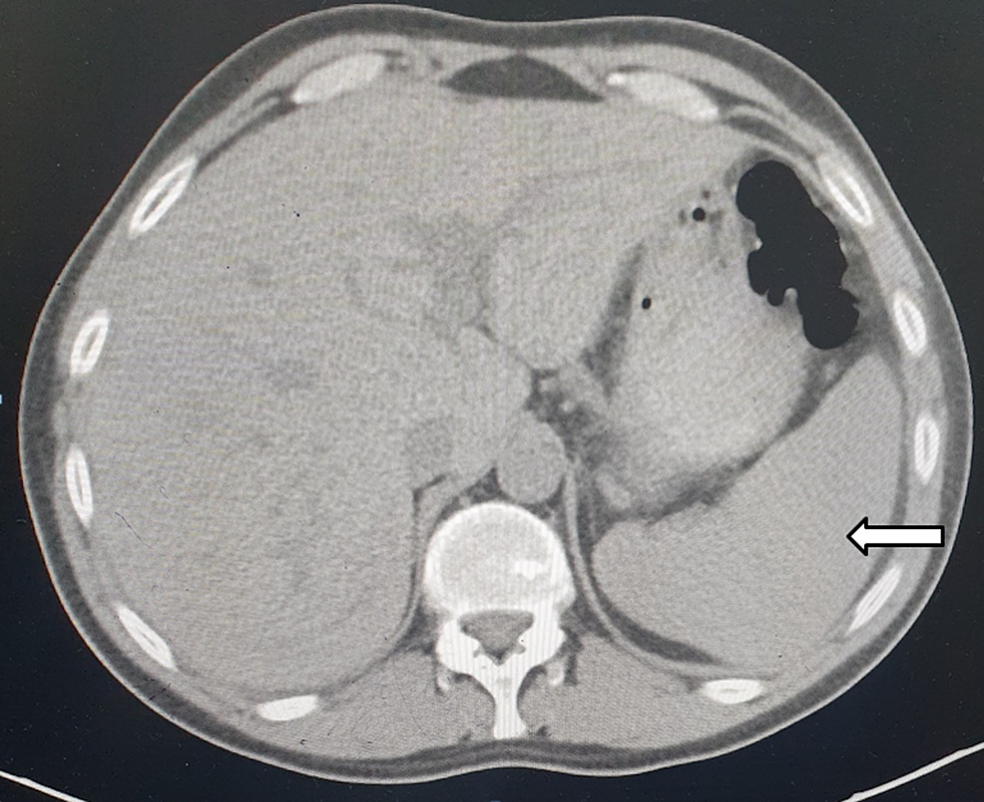
CT scan demonstrating splenomegaly (white arrow)

Computerized tomographic scans of the chest, abdomen, and pelvis revealed mild pericardial effusion (Figure 1) and splenomegaly (Figure 2). Pericardial effusion aspiration showed acute mixed inflammatory cells with predominant neutrophils, negative malignant cells, and no evidence of macrophage activation syndrome (MAS). Bone marrow aspiration results showed normocellular bone marrow with normal maturation and differentiation cells and no blast cells.

The diagnosis of AOSD was made based on Yamaguchi criteria; he had four major criteria: fever, arthralgia, typical rash, and neutrophilic leukocytosis, and two minor criteria, splenomegaly and negative ANA and RF. Then, the patient was started on methylprednisolone 1gm daily for three days, and he showed significant improvement in his symptoms. Therefore, he was discharged home on prednisolone 30mg daily and methotrexate 25mg weekly. Two months later, he presented complaining of mild shortness of breath; TTE showed circumferential pericardial effusion (maximal diameter, 13 mm) without cardiac collapse, so tocilizumab 600mg every four weeks was added. Four months later, he presented for his follow-up visit where he was asymptomatic, and his follow-up TTE showed no pericardial effusion. The patient continued to show improvement on prednisolone, tocilizumab, and methotrexate and was maintained on them.

## Discussion

**Figure 3:**
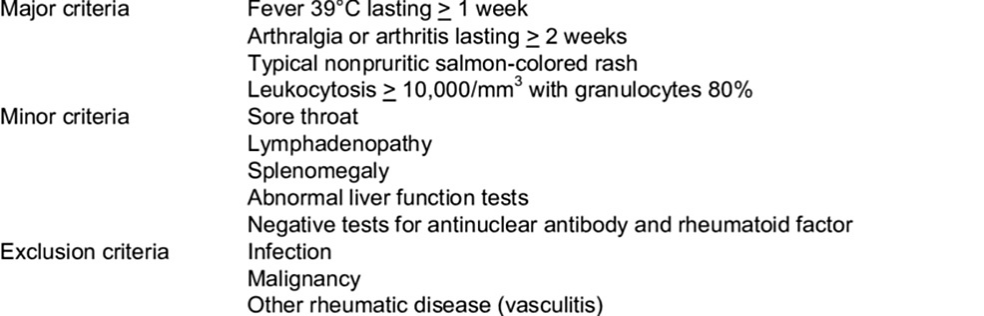
Yamaguchi criteria for AOSD diagnosis AOSD - adult-onset Still's disease

AOSD is a rare inflammatory disease that affects multiple organ systems. Patients between the ages of 16 and 35 are most affected [6,7]. The main cause of this illness remains unclear. Possible contributing factors are genetic predisposition, viral and bacterial infections, neoplasms, and inflammatory processes [8,9]. AOSD is characterized by prolonged fever, a salmon-colored pink rash, arthritis, and other systemic manifestations. For example, lymphadenopathy, polyserositis, interstitial lung disease, and hepatitis [10]. Pericarditis is one of the common cardiac manifestations of AOSD that can be complicated by pericardial effusion and rarely by cardiac tamponade [3,11]. However, as a rare complication, cardiac tamponade can be the initial presentation of AOSD [11]. AOSD is diagnosed by applying Yamaguchi criteria (Figure 3) and ruling out other infectious, neoplastic, and autoimmune causes [4]. Multiple cytokines like interleukins (IL-1 and IL-6) are involved in the pathogenesis of AOSD, making biologic drugs that target interleukins an evolving treatment option [4]. Corticosteroids is the first-line management of AOSD; other options include methotrexate and other DMARDs, anakinra (IL-1 inhibitor), tocilizumab (IL-6 antagonist), and tumor necrotic factor blockers [12,13].

Our patient presented to our hospital with a picture of cardiac tamponade that was confirmed by TTE, followed by emergency pericardiocentesis. He also complained of intermittent spiking fever, bilateral joint pain in his hands, knees, and ankles, and pink rash on his arms for about three weeks before his presentation. On physical examination, a salmon-colored rash was seen over his upper limbs, and he was found to have splenomegaly. Initial evaluation showed neutrophilic leukocytosis, high inflammatory markers, hyperferritinemia, positive D-dimer, negative ANA and RF.

The diagnosis of AOSD was made by applying the Yamaguchi criteria. The patient tremendously responded to pulse methylprednisolone, and he was successfully treated with Prednisolone, Methotrexate, and Tocilizumab as an outpatient. Six months later, his follow-up TTE revealed no pericardial effusion, and the patient significantly improved.

## Conclusions

AOSD is a rare inflammatory disease that can be rarely complicated by cardiac tamponade. Nevertheless, our case provides evidence that AOSD should be considered in the differential diagnosis of cardiac tamponade. It also demonstrates that the treatment of AOSD has a good outcome and can effectively prevent potentially lethal complications like cardiac tamponade.
